# MR1-Restricted T Cells Are Unprecedented Cancer Fighters

**DOI:** 10.3389/fimmu.2020.00751

**Published:** 2020-04-28

**Authors:** Alessandro Vacchini, Andrew Chancellor, Julian Spagnuolo, Lucia Mori, Gennaro De Libero

**Affiliations:** Experimental Immunology, Department of Biomedicine, University of Basel and University Hospital Basel, Basel, Switzerland

**Keywords:** MR1, MR1T, self-antigens, tumor recognition, T-cell therapy

## Abstract

Non-polymorphic MHC class I-related molecule MR1 presents antigenic bacterial metabolites to mucosal-associated invariant T (MAIT) cells and self-antigens to MR1-restricted T (MR1T) cells. Both MR1-restricted T cell populations are readily identified in healthy individuals, with MAIT cells accounting for 1–10% of circulating T cells, while MR1T cells have frequencies comparable to peptide-specific T cells (<0.1%). Self-reactive MR1T cells display a heterogeneous phenotype, and are capable of releasing both T_H1_ and T_H2_ cytokines, supporting not only activation of inflammation but also contributing to its regulation. Importantly, MR1T cells recognize and kill a diverse range of MR1-expressing tumor cells. On the other hand, evidence suggests MAIT cells augment cancer growth and metastases. This review addresses the potential role of MR1-restricted T cells in controlling tumor cells, facilitating their elimination and regulating cancer immunity. We also discuss therapeutic opportunities surrounding MR1-restricted T cells in cancer.

## Introduction

T cells restricted for antigen presenting molecules with limited or absent polymorphism, like cluster differentiation 1 (CD1) family of proteins or MHC class I-related molecule 1 (MR1), respectively, comprise adaptive-like T cells participating in immune homeostasis and in diseases (1). They express T cell receptors (TCRs) that sense non-peptide antigens and constitute discrete populations expressing either TCR αβ or γδ heterodimers. These TCRs can either be diverse or have strong expression bias being semi-invariant (1–3), such as in the case of mucosal-associated invariant T (MAIT) cells (4, 5). Since their discovery, major efforts have been made in exploring the role of MAIT cells in disease settings such as infection, autoimmunity and cancer. Due to the lack of human studies, understanding of MR1-associated antigens and mechanisms behind MR1 presentation, progress has been slow and sometimes inconclusive. More recently, a diverse, self-reactive, TCR αβ T cell population was identified which was MR1-restricted and exhibited anti-tumor responses (6). Seemingly present in all healthy donors tested, they represent an excellent candidate for further study in cancer. This review will discuss current knowledge regarding the roles of MR1-restricted T cells in cancer and their possible introduction in cell therapy.

MR1 is highly conserved among most mammals, in particular the α1 and α2 domains have 90 and 89% homology between mice and humans, respectively (7, 8). Furthermore, the MR1 sequence is the same between different individuals, and, as such, is termed non-polymorphic (9). The MR1 gene is encoded in chromosome 6 (8) and is ubiquitously transcribed in all human cells. The structural features of MR1 are very similar to that of MHC class I. A cleft between the α1 and α2 domains containing α-helices form a solvent exposed antigen-binding pocket atop a β-pleated sheet in a manner dissimilar to that of the narrow channels formed in the CD1 family. However, instead of a lining of polar residues in the case of MHC class I or indeed hydrophobic residues like CD1, the MR1 pocket is lined with largely aromatic residues to confer an environment that is both hydrophobic and charged (10). These features endow MR1 with the space and capacity to bind diverse small cyclic and bicyclic molecules (11). Moreover, the MR1 pocket seems not to accommodate peptide chains, nor does it exhibit open ends like MHC class II (10–12).

The first MR1 ligands to be characterized were vitamin B metabolites, in particular a metabolite of folic acid (vitamin B9) called 6-formylpterin (6-FP) and precursors of riboflavin (vitamin B2) as 6,7-dimethyl-8-D-ribityllumazine (RL-6,7-diMe) and 5-(2-oxopropylideneamino)-6-D-ribitylaminouracil (5-OP-RU) (10, 12). Later studies identified riboflavin itself and riboflavin adducts 7,8-didemethyl-8-hydroxy-5-deazariboflavin (FO), photolumazine I (PLI) and photolumazine III (PLIII) (13). *In silico* modeling allowed the discovery of further MR1-presented small molecules including: 3-formylsalicylic acid and diclofenac metabolites that were responsible for MAIT inhibition and weak activation of rare MAIT TCR, respectively (11). Furthermore, other studies implied bacterial antigens other than riboflavin metabolites (14) as well as tumor-associated antigens (1, 15). Therefore, the pocket of MR1 is highly plastic and might allow binding of other unknown antigens. Interestingly, all known antigens bind the A'-pocket leaving the F' unfilled. As the F' pocket is shared among MR1 molecules from different species, its evolutionary conservation suggests an important role. Although it could be possible that the F' pocket plays an important role in MR1 refolding and proper trafficking within the cell, like MHC class I molecules binding to tapasin and tapasin-related molecules, or MHC class II molecules binding to the invariant chain, there is the possibility that it can accommodate undiscovered ligands that are bigger than the small antigenic metabolites identified so far.

**Figure 1:**
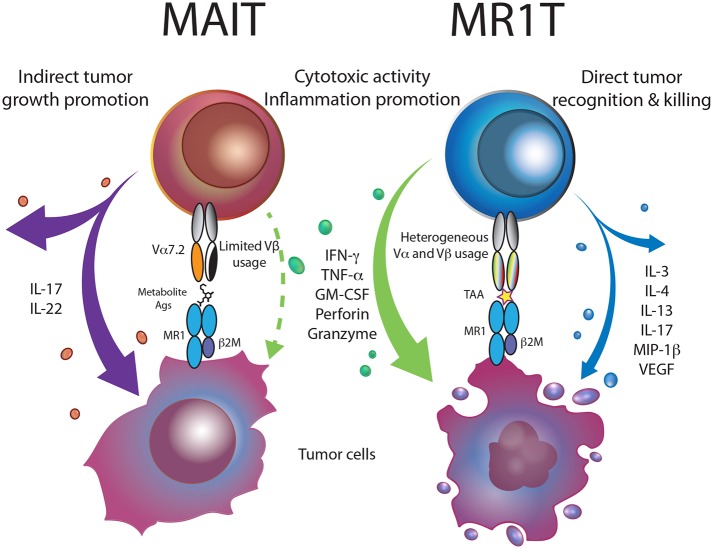
MR1-restricted T cells in cancer. Bacterial metabolite-reactive MAIT cells, within the tumor microenvironment, are skewed toward the production of Th17 cytokines, promoting tumor growth and metastasis. MR1T cells recognizing MR1-presented tumor-associated antigens (TAA), release a vast array of cytokines and kill tumor cells, thus supporting cancer immunity.

MAIT cells classically express a Vα7.2-Jα33 (TRAV1-2-TRAJ33) TCR, paired to a limited number of β chains for example Vβ2 (TRBV20) or Vβ13 (TRBV6) (Figure 1) (4, 5, 16, 17). Alternative TRAJ genes are also used when maintaining a CDR3α loop conserved in length and with a Tyrosine in position 95, crucial for 5-OP-RU recognition (18). Furthermore, atypical TRAV1-2^−^ MAIT cells have been described, that are stained with a 5-OP-RU-loaded MR1 tetramer and react to bacteria-infected cells (14, 19). In contrast to MAIT cells, MR1T cells are a novel population of self-reactive MR1-restricted T cells that are characterized by diverse TCR usage and are not stimulated by bacterial ligands (6, 20). MAIT cells have a very high frequency (1–10%) in the blood of healthy individuals (21, 22) compared to MR1T cells that are less abundant and found at a frequency of ~1:2500 of circulating T cells (6). Regarding localization, MAIT cells are enriched within barrier tissues and in particular in mucosa, gut lamina propria, liver (16, 17, 23, 24), lungs and skin (25, 26) and less frequently in lymph nodes (23). Less is known about MR1T cells except that they were found in the blood of each healthy individual studied and MR1T cell clones were activated by cancer cell lines in an MR1-dependant manner (6, 20).

Development of MAIT cells is thought to occur after recognition of commensal bacteria-derived antigens presented by double-positive (DP) thymocytes (23, 26–28). A three-stage transcriptional program drives MAIT cells to acquire an innate-like phenotype, characterized by high expression of CD161 and transcription factors PLZF, T-bet and RORγT (21, 27, 29–31). Up to five different subsets of MAIT cells can be distinguished in humans based on the expression of TCR co-receptors. The most abundant subset in human blood consists of CD4^−^CD8αβ^+^ or CD8αα^+^ cells (approximately 80% of MAIT cells); double-negative (DN) CD4^−^CD8^−^ represent about 15% of total MAIT cells, few CD4^+^CD8^−^ and CD4^+^CD8^+^ are present (12, 30). So far, the analysis of a large number (>100) of MR1T cell clones showed that they were either CD8^+^ or DN (our unpublished studies) and only few of them expressed CD161 (6), suggesting that these cells are heterogeneous. MR1T cell functional heterogeneity is even more pronounced, with different clones displaying distinct T_H1_, T_H2_, or T_H17_ cytokine and transcriptional profiles upon stimulation (Figure 1) (6). MAIT cells do not express the lymph node-homing receptors CCR7 and CD62L, and only slight differences were observed in their expression of chemokine receptors and integrins, that dictate their likelihood for tissue residency (23, 30, 32). MR1T cells also show tissue-homing capacity, but lower expression of the chemokine receptors CCR4 and CCR6, compared to representative MAIT clones (6), suggesting comparatively different localization patterns.

MAIT cell activation occurs after TCR engagement with MR1-presented antigens on infected cells (33), as well as in a TCR-independent manner after stimulation by inflammatory cytokines such as IL-12 and IL-18 or type I interferons (34–37). Immediately after antigen recognition and activation, MAIT cells have the capacity to release granzyme B and perforin to promptly kill infected cells (17, 23, 24). In addition, T_H1_ and T_H17_ cytokines are secreted such as IFNγ, TNFα, IL-2, IL-17 that function against invading pathogens (26, 36, 38, 39). Sensitivity to cytokines underlies MAIT cells' ability to tailor their response to different scenarios despite bearing a semi-invariant TCR (40). Uncoupled TCR and cytokine stimulation induce distinct effector functions on MAIT cells, that become synergistic when occurring at the same time (36, 37). TCR engagement in the absence of cytokines induces homeostatic, tissue repair-oriented functions that instead, in presence of cytokine co-stimulation, become pro-inflammatory exerting an anti-microbial activity (36, 39). Cytokine modulation of MAIT cell function impacts the role of these cells in different pathological conditions, as discussed below.

## Mait Cells in Pathology

Several studies have addressed the relevance of MAIT cells in infectious disease, autoimmunity and cancer, both in humans and in mouse models. The frequency of MAIT cells in the blood is often reduced in patients suffering of bacterial or viral infections, autoimmune or metabolic diseases and several cancers, compared to that of healthy donors or before disease onset (41–47). A reduction in circulating MAIT cell frequency reflects their migration toward peripheral tissues, where MAIT cells are increased or redistributed in the proximity of inflamed areas. This is largely driven by the expression of several tissue-homing chemokine receptors and integrins (and absence of lymph node-homing receptors) whose expression is increased by inflammatory cytokines (34–36). Cytokine-dependent, TCR-independent activation of MAIT cells becomes prominent in the case of viral infections and autoimmune-diseases, where bacterial antigens are absent at the pathologic site. In addition, MAIT cells are necessary to maintain homeostatic conditions, mucosal barrier integrity and play beneficial roles in the context of acute bacterial infections (33, 48, 49). On the other hand, MAIT cells were found to be deleterious in chronic bacterial infections (50, 51) and a variety of autoimmune diseases where their effector functions are altered (43, 44, 49, 52–56). Therefore, finding unifying rules regarding the role of MAIT cells in disease is difficult, and is likely dependent on the local cytokine milieu, other immune cell populations and microbial species present (46, 47).

## MAIT Cells in Cancer

The possibility of directing the host immune system against tumor cells is the rationale behind immunotherapy which has largely focused on local T cell activation by checkpoint blockade inhibitors (57, 58) or redirecting the specificity of immune cells against tumors (59). Many resources have been used in attempt to unleash the antitumor potential of T cells, whose infiltration inside the tumor positively correlates to a good prognosis (60, 61). Given the possibility to overcome the limitations of extensive HLA polymorphism by exploiting monomorphic MR1 molecules, priority should be given to understand the roles of MR1-restricted T cells in cancer.

While the presence of tumor-reactive MR1T cells in healthy or pathologic tissues has not been assessed yet, MAIT cell frequencies are altered in tumors (61). As observed for other diseases, MAIT cells seem to be subjected to modifications in numbers and phenotype in cancer. For example, in colorectal cancer, different studies show either similar, or decreased frequencies of peripheral blood MAIT cells in patients compared to healthy individuals (45, 62, 63). Nevertheless, MAIT cells were found to be increased in primary colorectal lesions and hepatic metastatic lesions, compared to unaffected tissues (45, 62, 64, 65). The phenotype of MAIT cells in the tumor niche is also altered, since they produce less IFN-γ (45, 62) (Figure 1), where MAIT cell infiltration correlated with a poor survival (64). As for liver-related diseases, MAIT cells accumulate locally (66–69), while their frequency decreased in the blood of hepatocellular carcinoma (HCC) patients, compared to healthy donors (68, 69). Indeed, within HCC tumor lesions, MAIT cell frequency was reduced compared to peritumoral hepatic area and the HCC microenvironment polarized tumor-infiltrating MAIT cells to an exhausted phenotype (PD-1^high^ CTLA-4^+^ TIM-3^+^), leading to a reduced response to bacterial antigens and the production of tumor-promoting cytokines (69). Therefore, evidence suggests MAIT cells have a tumor-supporting function and their presence is associated with an unfavorable outcome (69).

A similar observation was made in multiple myeloma (MM), where MAIT cells in newly diagnosed MM patients have reduced effector functions and CD27 expression, indicative of a functionally impaired phenotype (70). MAIT cells were able to expand in response to gastrointestinal microbiota and promote gut integrity only after bone marrow transplantation (71, 72). Reconstitution of the MAIT cell population resulted in suppression of conventional T cell proliferation and prevention of graft-*vs*.-host disease, indicating a possible regulatory role for MAIT cells (71–73). This immune-suppressive regulatory role of MAIT cells was also recently described in mouse cancer models where MAIT cells promoted progression and metastases of melanomas and methylcholanthrene-induced fibrosarcoma (74). In these models, MAIT cells activated *in vivo* by MR1-expressing tumor cells suppressed NK cells effector functions, chiefly through IL-17A secretion (74). MAIT cell activation was blocked by treatment of tumor cells with 6-FP or administration of MR1 blocking antibodies, suggesting TCR-mediated activation of MAIT cells. Considering that in the described models tumor cells presented MAIT antigens (74), MAIT cells could also induce tissue-repair and perhaps tumor-promoting functions if TCR engagement was solely responsible for activation (36, 39). In sum, MAIT cells often accumulate within tumors and under the influence of the tumor microenvironment, acquire an exhausted phenotype and skewed effector functions to suppress immune anti-tumor functions (Figure 1).

## MR1T Cells in Cancer

Self-reactive MR1-restricted MR1T cells were isolated and identified by their ability to recognize tumor cells in a TCR-mediated manner. Given their heterogeneous functional profile, they could have a variety of functions and modulate the innate and adaptive responses to cancer. MR1T cells were originally described as capable of recognizing several tumor cell lines from different tissues expressing physiological levels of MR1 (6), thus revealing an unexpected broad crossreactivity toward tumor cells. A recent study confirmed this finding and by investigating one TCR showed the capacity of this T cell clone to kill several tumor cell lines (20). Such broad crossreactivity provides the bases for potential use of MR1T cells in immunotherapy of different tumors as recently discussed (75).

In contrast to MAIT cells, MR1T cells do not recognize bacteria-infected cells, and are not activated by healthy cells (6, 20). The self-antigens responsible for activation are as yet unknown, but they were shown to be displaced from MR1 by vitamin B derivatives (5-OP-RU and acetyl-6-formylpterin), indicating that they exploit the same binding pocket as of other MR1 ligands (6, 20). So far, evidence indicates that these hydrophilic compounds can be found in either freshly explanted mouse breast tumor or from lysed THP-1 cells (6). Evidence strongly suggests the presence of multiple self-antigens and antigen-specificity of MR1T cells, since two T cell clones bearing different TCRs distinguished between two different lysate fractions (6). Furthermore, a recently described MR1T clone is selectively activated by wild-type MR1 and not by the K43A mutant (20), possibly indicating that some of the antigens recognized by MR1T cells could covalently bind MR1 on lysine 43 as already described for 5-OP-RU (12). Alternatively, the lysine 43 mutant does not assume the conformation required for the stimulation of the single clone described by Crowther et al. In conclusion, unlike MAIT cells, MR1T cells are activated by MR1-bound self-antigens expressed by tumor cell lines grown *in vitro* or *in vivo*.

Given the recognition of multiple cancer cells lines by individual MR1T cell clones, it is feasible that cancers share common antigens presented by MR1. The frequency and the phenotype of MR1T cells in cancer patients are not yet known, however, given their ability to recognize *in vivo* expanded tumor cells, their role might be beneficial to patients. Considering the aforementioned features and the possibility to confer tumor reactivity to other T cells by TCR transfer (6, 20), MR1T cells represent potential candidates for broadly reactive T cell therapy in cancer (75). The functional dichotomy of MAIT and MR1T cells and their potential role in cancer immunity are outlined in Figure 1.

## MR1-Restricted T-Cell Therapies: Current Opportunities and Future Perspectives

Three attributes are required for off-the-shelf anti-tumor T-cell therapies; (i) specificity toward antigens expressed by tumor and not normal cells; (ii) pan-recipient compatibility to avoid host-*vs*.-graft responses in infused patients, and (iii) availability of TCRs that recognize different tumor types.

Biochemical studies are required for the identification of antigens presented by MR1 and generated in tumors but not in normal cells. Understanding their nature will reveal the molecular basis of their apparent accumulation in tumor cells. In addition, these antigens will be ideal tools for the generation of MR1 tetramers to further study MR1T cells. Furthermore, TCR structural motifs that confer MR1-restriction will also be of relevance. Investigating large numbers of MR1-restricted TCRs will perhaps provide this type of information and will be key for engineering of pan-cancer MR1T cells for off-the-shelf therapy.

An intrinsic and optimal feature of TCR restriction to non-polymorphic antigen-presenting molecules is what can be defined as pan-recipient compatibility. Indeed, these TCRs are devoid of graft-*vs*.-host activity, which instead prevents the therapeutic use of HLA-restricted TCRs in the entire population. This feature opens the possibility of transferring the same MR1T TCRs to every patient, overcoming the need of isolating HLA-matched tumor-specific TCR for every recipient. Moreover, the non-polymorphic nature of MR1 allows the generation of engineered HLA-deficient MR1T cells for an off-the-shelf therapy, immediately available for on-demand infusion, also in patients with compromised T cell compartments. There is also added value of using such TCRs that are seemingly not influenced by the expression of CD4 or CD8 co-stimulatory molecules, thus redirecting responses in both CD4^+^, CD8^+^ and DN T cells required for efficient cytotoxic T cell priming and cancer immunity (76–78).

Off-the-shelf use may also require a combination of TCRs that recognize the antigens preferentially expressed in each individual tumor. Therefore, a novel classification of tumors according to their accumulation of MR1-presented antigens and their screening in each patient will represent a method to select the MR1T TCRs with relevant specificity in a personalized immunotherapy.

Other important issues are the functions of MAIT cells and of MR1T cells with respect to their direct role in tumor immunity. In mice, the MAIT_17_ subset is more prominent than MAIT_1_ and although MAIT cells in humans have a mixed T_H1_-T_H17_ phenotype (79–81), in the case of cancer and other pathologies it can be skewed toward the production of the IL-17, a multi-functional cytokine that can support tumor growth (82–84). According to this evidence, potential therapies to block MAIT cell activity in cancer have been proposed. These proposals converge on the possible blockade of MR1 presentation rather than targeting MAIT cells, given their important role in the homeostasis of mucosal tissues against invading pathogens. Infusions of MR1-inhibitory ligands such as acetyl-6-FP or MR1-blocking antibodies could indeed block MAIT cell activation, but it is not known whether this could increase gut permeability as observed in the case of MR1-deficient NOD mice (49). There are two additional possible outcomes that detract from this strategy. The first, is that blocking MR1 would affect its antigen presentation to all MR1-restricted T cells, including tumor-reactive MR1T cells that likely have beneficial activity, stimulating rather than suppressing cancer immunity and directly killing tumor cells. Secondly, repeated infusions of MR1 ligands which do not stimulate MAIT cells, might lead to priming and expansion of other MR1-restricted T cells specific for the infused molecules, with potentially deleterious side effects.

A more appealing use of MR1 ligands might exploit drugs to increase the generation or the accumulation of MR1T antigens in tumor cells. This mechanism of action in immunotherapy has already been proposed with aminobisphosphonate drugs to increase tumor recognition by Vγ9Vδ2 T cells (85). Thus, characterization of the tumor-associated antigens recognized by MR1T cells will be important to decipher the regulation of metabolic pathways for pharmacological exploitation.

Discovery of these antigens could also stimulate the development of vaccines to expand and generate memory populations of tumor-reactive MR1T cells, a strategy discussed for other tumor-associated antigens (86). Broad tumor crossreactivity of MR1T TCRs (6, 20) will add value to this strategy and will represent an innovative type of tumor vaccination.

## Concluding Remarks

Antigen-presentation by MR1 functions to report the metabolic programming of microbes (87) and undoubtedly the host, to T cells. Our knowledge of MAIT cells has developed over the last decade but less is known about the more recently discovered MR1T cells. Further work to identify MR1T-stimulatory molecules and a broader understanding of MR1T cell diversity is needed to fully appreciate the relationship of antigen recognition and their physiological functions. So far, evidence indicates a pro-tumoral activity of MAIT cells, whereas the published literature on MR1T cells indicates they have an anti-tumoral activity. Thanks to these features, MR1T cells represent an interesting population to exploit in cancer immunotherapy.

## Author Contributions

All authors listed have made a substantial, direct and intellectual contribution to the work, and approved it for publication.

## Conflict of Interest

The University of Basel has filed patents on MR1T cells. The authors declare that the research was conducted in the absence of any commercial or financial relationships that could be construed as a potential conflict of interest.
